# Supplementary figures and images for: The Adaptor Protein UvSte50 Governs Fungal Pathogenicity of Ustilaginoidea virens via the MAPK Signaling Pathway
Source: J Fungi (Basel). 2022 Sep 11;8(9):954. doi: 10.3390/jof8090954 (PMC9503583; doi:10.3390/jof8090954)

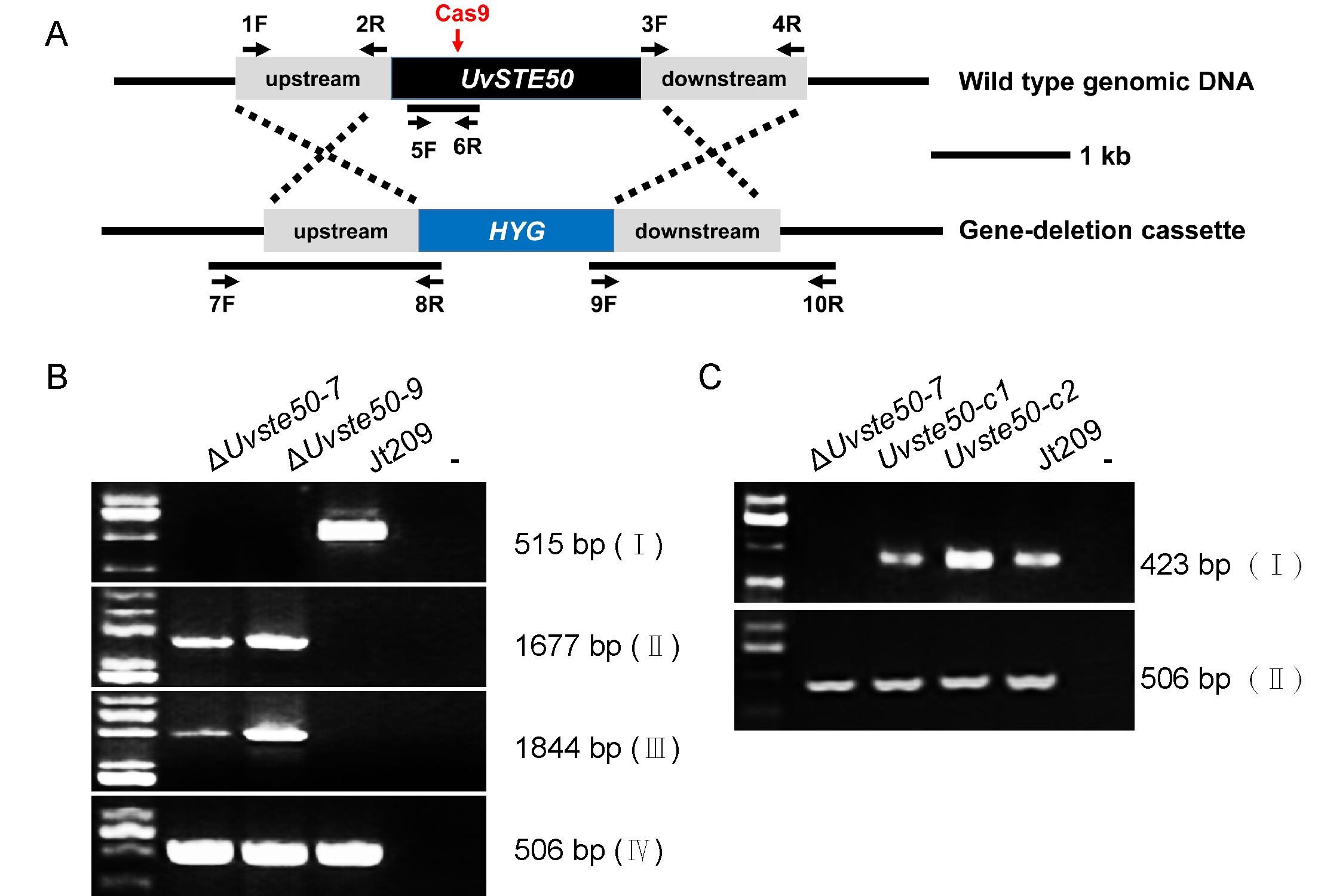

Supplement: Supplementary file 1 [file jof-08-00954-s001.zip › Figure S1.tif]

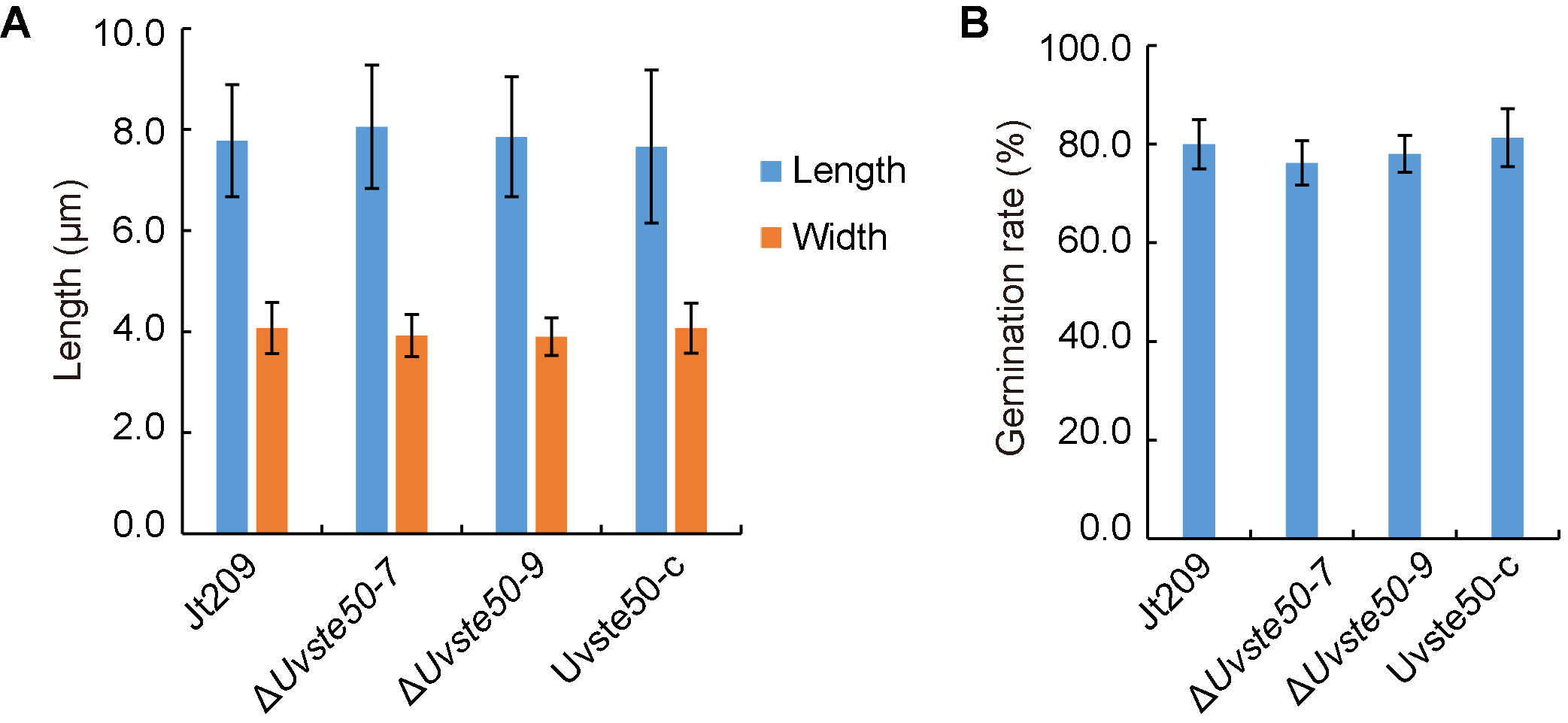

Supplement: Supplementary file 1 [file jof-08-00954-s001.zip › Figure S2.tif]

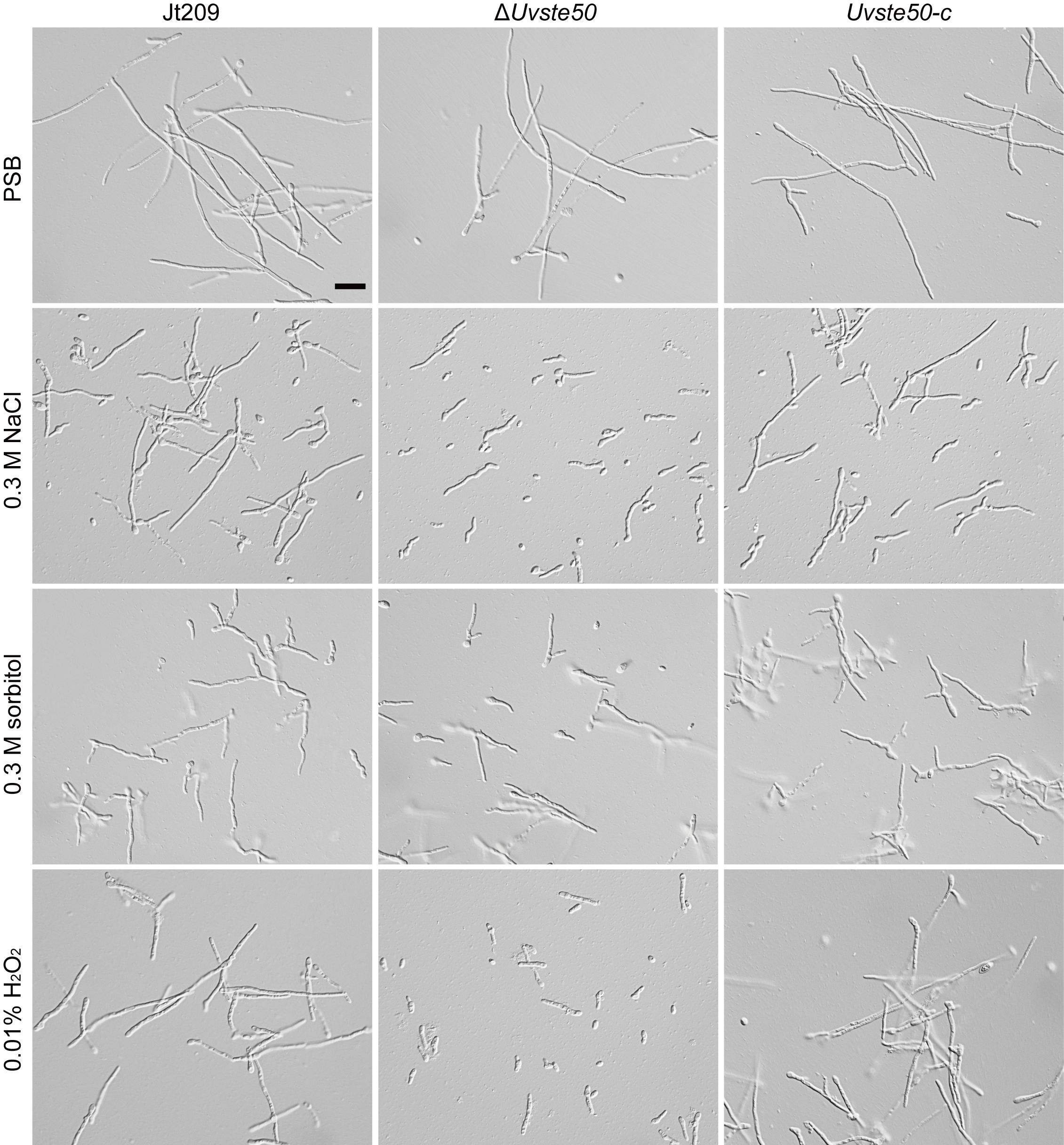

Supplement: Supplementary file 1 [file jof-08-00954-s001.zip › Figure S3.tif]

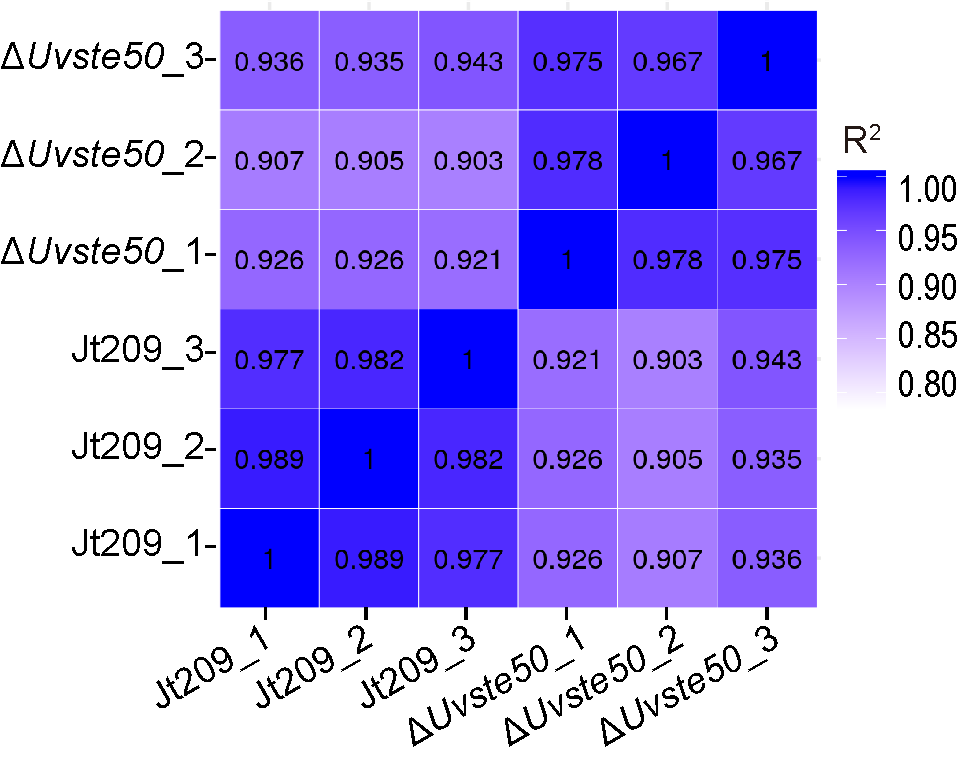

Supplement: Supplementary file 1 [file jof-08-00954-s001.zip › Figure S4.tif]

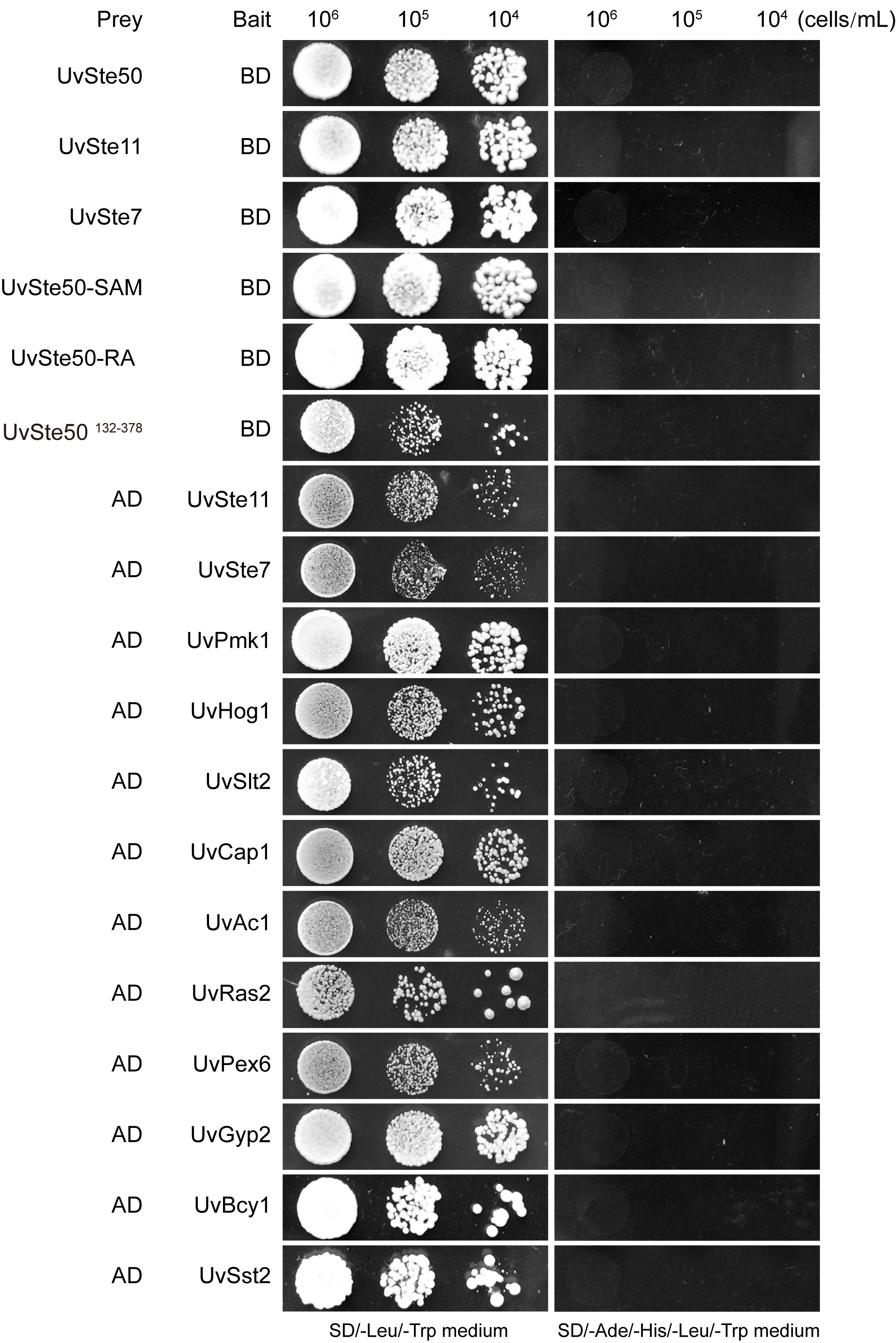

Supplement: Supplementary file 1 [file jof-08-00954-s001.zip › Figure S5.tif]

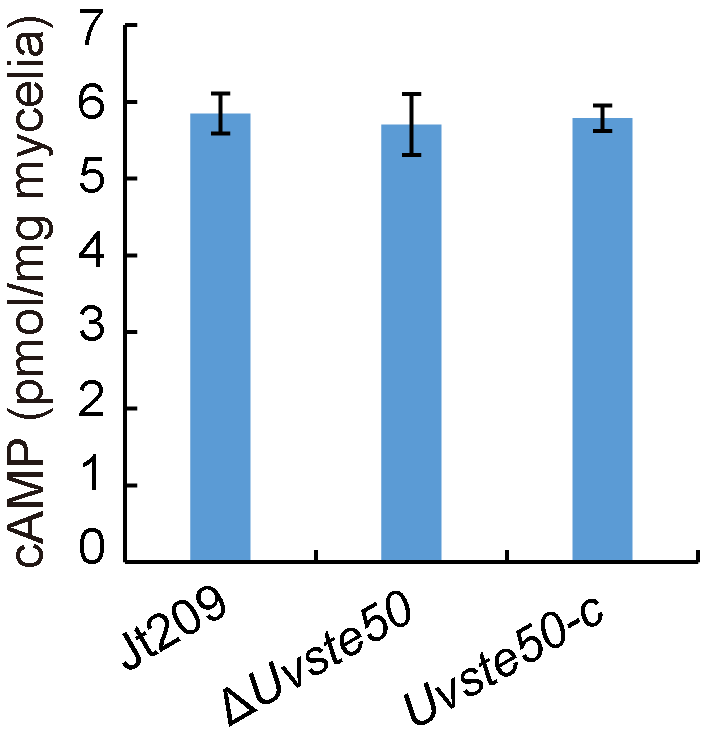

Supplement: Supplementary file 1 [file jof-08-00954-s001.zip › Figure S6.tif]
